# Animal and Plant Protein Oxidation: Chemical and Functional Property Significance

**DOI:** 10.3390/foods10010040

**Published:** 2020-12-25

**Authors:** Youling L. Xiong, Anqi Guo

**Affiliations:** Department of Animal and Food Sciences, University of Kentucky, Lexington, KY 40546, USA; anqi.guo@uky.edu

**Keywords:** protein oxidation, antioxidants, carbonyls, protein functionality, texture

## Abstract

Protein oxidation, a phenomenon that was not well recognized previously but now better understood, is a complex chemical process occurring ubiquitously in food systems and can be induced by processing treatments as well. While early research concentrated on muscle protein oxidation, later investigations included plant, milk, and egg proteins. The process of protein oxidation involves both radicals and nonradicals, and amino acid side chain groups are usually the site of initial oxidant attack which generates protein carbonyls, disulfide, dityrosine, and protein radicals. The ensuing alteration of protein conformational structures and formation of protein polymers and aggregates can result in significant changes in solubility and functionality, such as gelation, emulsification, foaming, and water-holding. Oxidant dose-dependent effects have been widely reported, i.e., mild-to-moderate oxidation may enhance the functionality while strong oxidation leads to insolubilization and functionality losses. Therefore, controlling the extent of protein oxidation in both animal and plant protein foods through oxidative and antioxidative strategies has been of wide interest in model system as well in *in situ* studies. This review presents a historical perspective of food protein oxidation research and provides an inclusive discussion of the impact of chemical and enzymatic oxidation on functional properties of meat, legume, cereal, dairy, and egg proteins based on the literature reports published in recent decades.

## 1. Introduction

Protein oxidation is a common occurring yet largely neglected phenomenon. Unlike lipid oxidation, which is readily detected by consumers due to volatile off-flavor compounds generated [1], protein oxidation usually occurs unnoticed. Nevertheless, through discovery research in the later part of the 20th century, mostly conducted in the field of health sciences, it has become clear that, similar to lipids, proteins are rather susceptible to reactive oxygen species (ROS). Previous studies at both the cellular and molecular levels had concentrated on the deleterious consequences of protein autooxidation in human health and age progression. Oxidation of cellular enzymes and structural proteins has been linked to Alzheimer’s disease (formation of amyloid plaques), chronical aging, Parkinson’s disease, cataracts, muscle dystrophy, and atherosclerosis [2]. In fact, one of the principal theories for human aging has been derived from free radical oxidation of cellular proteins and other substances [3]. Accumulated evidence in diet-related pathological research suggests that protein oxidation in food systems and the consumption of extensively oxidized meat could have a significant negative impact on the health of humans [4].

Several early investigations have shown that, as is in living tissues, ROS can cause food proteins to polymerize, degrade, and interact with other food components to produce complexes [5,6,7,8]. Subsequent research, including recent studies, has produced ample new evidence that ROS-induced physicochemical modifications could significantly alter the functionality (i.e., gelation, emulsification, foaming, film formation, and water-binding) of muscle [9,10,11,12,13,14,15], egg [16,17], dairy [18,19,20,21], legume [22,23,24,25], and cereal [26,27,28] proteins. Table 1 lists the proteins from different commodity food groups that have been widely subjected to oxidative studies. With the advent of sophisticated separation techniques combined with robust analytical instruments, for example, ultra HPLC, electron spin resonance, and advanced mass spectrometry, food scientists are now able to acquire new insights into the mechanism and understand many of the molecular processes involved in protein oxidation in food systems [29,30,31]. Continuing investigations around this topic have led to the further understanding that many of the oxidative processes may be controllable and manipulatable through antioxidative processing, packaging, and ingredient strategies [32,33,34]. Conversely, where protein oxidation is desirable for food product texture formation, appropriate oxidizing agents or processing conditions may be introduced to improve food product quality [35,36,37,38].

In food systems, protein oxidation can occur unprovoked (*in situ* autooxidation) or initiated by processing treatments, such as ultraviolet and ionizing radiations, photosensitization, and catalysis with exogenous redox enzymes. Oxidation typically involves free radical-induced modification of amino acid side chain groups (e.g., sulfhydryls and amines), peptide bonds, and intramolecular forces that maintain the spatial conformational structure of proteins [39]. Other oxygen species, such as secondary products of lipid peroxidation, can also induce protein oxidation by binding with reactive side chain groups [40]. Aggregation (or, conversely, fragmentation) occurs as a result of oxidative structural disruption which either impairs or improves the texture-forming and other functional properties of proteins in food [30,41]. To regulate protein oxidation for desirable food product attributes and palatability, antioxidants are increasingly utilized to produce optimum gelling, emulsifying, film-forming, and water-holding capacities.

In this paper, we aim to present a brief review of protein oxidation occurring in food systems and discuss the impact on the functionality and quality attributes of food products in the context of diverse food commodities, i.e., meat, milk, egg, legumes, and cereals. The specific conditions under which protein oxidation is investigated are highlighted, and potential antioxidative strategies for controlling and modulating protein oxidation are suggested. Hence, differing from previous reviews, the present review provides an inclusive coverage of collective food groups through generalized analysis as well as individualized accounts for protein oxidation and antioxidant strategies, making it scientifically and practically relevant in the field. It should be noted that, the potential impact of oxidation on nutritional quality of proteins and proteinaceous foods (which is generally indicated by altered protein digestibility and loss of essential amino acids) [42], will not be a focus in the present review. As well, pathological consequences of regularly consuming oxidized proteins will not be included in the present work as such information has been comprehensively described in a previous seminal paper [4].

## 2. Mechanisms of Protein Oxidation and Assessment

Protein oxidation in biological systems, including food, is rather complex and remains not well characterized. The process requires activated oxygen. The oxygen activation may be initiated by redox enzymes, photosensitizers, ultraviolet and ionizing radiations, or metal-catalyzed one-electron reductions to generate a wide range of ROS, including superoxide anion (O_2_^•−^), hydrogen peroxide (H_2_O_2_), and hydroxyl radical (^•^OH) [7,43]. Enzyme-catalyzed protein oxidation involves at least two steps: the catalytic production of specific ROS followed by their attack of proteins. Examples of oxidative enzymes of food relevance are glucose oxidase that produces H_2_O_2_ from glucose [44], laccases that generates phenolic radicals [45], lipoxygenase that catalyzes oxidative formation of hydroperoxides from unsaturated lipids [46], and lactoperoxidase that produces peroxyl radicals [47].

Photochemical oxidation occurs via the singlet oxygen (^1^O_2_) pathway in which molecular oxygen in the stable triplet state is activated by photosensitizers, such as riboflavin, protoporphyrin, and chlorophyll [21,48]. The radical intermediates of ^1^O_2_ are reactive with many amino acid side chain groups to initiate protein oxidation. Direct oxidative reactions of proteins can be caused by UV radiations due to the absorption of tryptophan and tyrosine in the wavelength range of 280–325 (UVB) or 315–400 nm (UVA) [49]. This leads to electron transfer and hydrogen abstraction from proteins. On the other hand, when proteins are under ionizing radiations, such as γ-irradiation, radicals generated by the radiation, including ^•^OH and O_2_^•−^, will cause damage and modify the molecular properties of proteins [7,50].

**Figure 1:**
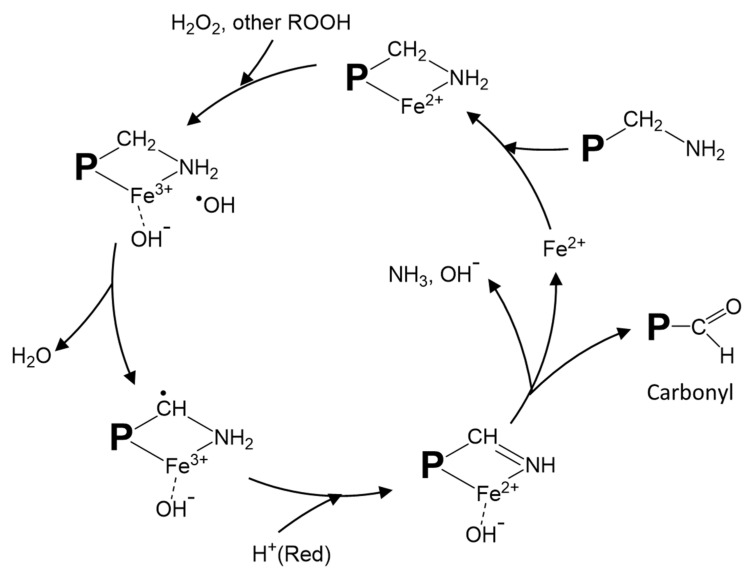
Proposed site-specific metal-catalyzed oxidation of protein amino acid residues. The illustration shows iron-catalyzed radical formation and carbonyl generation from oxidized lysine residues. A modified drawing from [51]. P: protein; ROOH: peroxide; Red: reducing compound.

Metal-catalyzed protein oxidation, which has been most extensively studied due to its wide occurrences, has its unique mechanism. During oxygen activation, the sequential reduction of O_2_ to O_2_^•−^ by Fe^2+^ (or vice versa, oxidation of O_2_^•−^ to O_2_ by Fe^3+^) to produce ^•^OH is exemplified by Haber–Weiss reaction (O_2_^•−^ + H_2_O_2_ → ^−^OH + ^•^OH + O_2_). Alternatively, Fe^2+^ can directly react with H_2_O_2_ to produce ^•^OH through Fenton reaction (Fe^2+^ + H_2_O_2_ → Fe^3+^ + ^−^OH + ^•^OH). Protein oxidation proceeds with the initial modification of amino acid side chain groups by the ROS generated and the conversion to carbonyl and other derivatives [2,43]. Stadtman and Levine [51] proposed a site-specific metal-catalyzed protein oxidation model in which loosely-bound transition metal ions, such as Fe^2+^ and Cu^+^, react with H_2_O_2_ to form ^•^OH that subsequently attacks amino acid side chains (Figure 1). According to this mechanism, the complex formed by the binding of Fe^2+^ to ε-amine group in lysine can react with H_2_O_2_ to produce ^•^OH through a Fenton-like pathway leading to ultimate production of carbonyls and other products. Other amino acid residues that can bind with Fe^2+^ to generate ^•^OH and ferryl ion include proline, histidine, arginine, and cysteine [52]. Protein radicals, which have a long half-life, could form via a similar radical-transfer mechanism [31].

In describing ROS-induced amino acid side chain modification, Statdman and Berlett [53] presented the relative reactivity of different amino acids. Virtually all amino acid residues are prone to ROS attack, but the most susceptible ones are sulfur-containing (cysteine and methionine) and amine-containing (arginine, lysine, and histidine) residues and those with bulky side chain groups, such as tryptophan, leucine, and phenylalanine (Table 2). Of special note is the oxidative conversion of cysteine to cystine (protein cross-linker), sulfanic acid, sulfinic acid, and sulfonic acid [54]. α-Aminoadipyl semialdehyde (from arginine and proline), γ-glutamyl semialdehyde (from lysine), and methionyl sulfoxide (from methionine) are examples of protein oxidation products that have been detected. In addition to side chain modification, ROS can attack protein peptide bonds producing protein fragments. Such cleavage is initiated by α-hydrogen abstraction to form a carbon-centered radical which subsequently reacts with O_2_ to produce the peroxyl radical adduct. The ensuing reaction with other ROS leads to the formation of alkyl peroxide and ultimately, alkoxy radical, setting the stage for peptide bond cleavage [55].

The formation of carbonyls (aldehydes and ketone) is a common consequence of protein oxidation. Protein carbonyls can form via direct modification of amino acid side chains by ROS, peptide bond cleavage, or through the adduction of non-protein carbonyl units. For example, ROS could directly attack lysine, arginine, proline, and threonine [51] to generate carbonyls. Amino acids with nucleophilic groups, such as histidine, cysteine, and lysine, can also be indirectly carbonylated by covalent binding with non-protein reactive carbonyl species, such as 4-hydroxy-2-nonenal (4HNE) and malondialdehyde (MDA), through Michael addition [56,57]. In addition, reducing sugars and Strecker degradation aldehydes can form complexes with proteins to generate protein-bound carbonyls [56]. Hence, by binding to proteins, these lipid- and carbohydrate-derived carbonyls contribute to the total carbonyl content in oxidized proteins.

As carbonylation occurs in almost all oxidized proteins, carbonyl is considered to be a reliable marker for protein oxidation. Carbonyl analysis, therefore, has become a common approach to estimating the extent of oxidative modification [58]. The method, initially developed by Brady and Elsmie [59] for aldehyde and ketone analysis, involves the derivatization of protein carbonyls with 2,4-dinitrophenylhydrazine (DNPH) to form the dinitrophenylhydrazone complex (Reaction (1)), which can be detected directly by spectrophotometry, HPLC, and mass spectrometry [60,61]. To improve the test sensitivity, immunoblotting or ELISA with an antibody against 2,4-dinitrophenyl can be used [62].


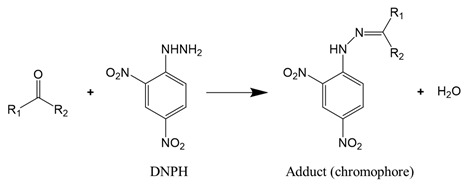
(1)

For proteins that have a significant number of cysteine or lysine residues (which are susceptible to oxidants), sulfhydryl and amine content analysis proves to be a valuable means to assess protein oxidation. The two functional groups can be quantified by the reaction with, respectively, Ellman’s reagent 5,5-dithio-bis-(2-nitrobenzoic acid) (DTNB) and 2,4,6-trinitrobenzene sulfonic acid (TNBS). The reaction of DTNB with free sulfhydryls produces a yellow chromophore (5-thio-2-nitrobenzoate, TNB) that absorbs strongly at 412 nm (Reaction (2)), while the reaction of TNBS with primary amines yields an orange-colored chromogenic product that can be readily measured at 335 nm (Reaction (3)).


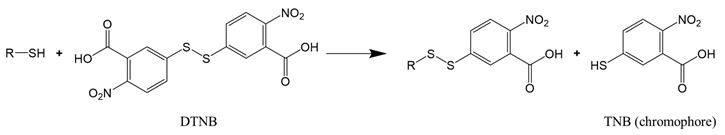
(2)


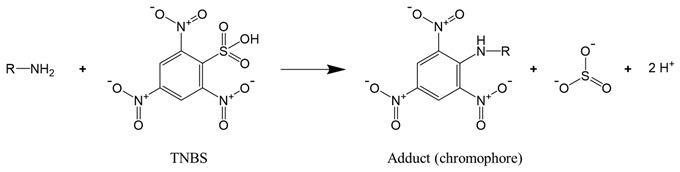
(3)

## 3. Protein Oxidation and Functionality Changes

### 3.1. Muscle Proteins

Oxidative changes occurring in raw and processed muscle foods are the most widely investigated compared with other food commodity systems. The research began in early 1990s when scientists discovered that protein oxidation, previously not recognized or detected, could readily occur in muscle foods, including land animal species (pork, beef, and poultry) and fish [14,63,64,65]. Those studies showed significant amino acid damage and structural changes in muscle proteins, especially myofibrillar protein (MP), when meat and fish were exposed to oxidizing environments. The oxidation modification, usually not affecting the flavor, was believed to have a causative role in textural quality of processed muscle foods. The loss of amino acids possessing susceptible side chain groups is the most notable result of oxidative change, and hydroxyl radical and fatty acid peroxyl radical species are commonly involved [41]. This is because the redox environment in disrupted muscle tissue, resembling reperfusion (oxygen restoration) of ischemic (hypoxic) or anoxic tissue in humans and animals [66] favors the production of ROS due to the abundance of their precursors and oxidation catalysts. Specifically, the remarkable susceptibility of muscle proteins to oxidation stems from the high concentrations of endogenous prooxidative transition metals (e.g., iron and copper), hemes, polyunsaturated fatty acids (PUFA), as well as molecular oxygen that is introduced into meat during processing [39,40]. As reported by Park and Xiong [67], in an iron/ascorbate-catalyzed oxidizing system, cysteine, methionine, and tyrosine in MP were the most susceptible amino acids; in a linoleic acid/lipoxidase oxidizing system, both sulfur-containing amino acids were modified; and in a metmyoglobin-oxidizing system, alanine, glycine, histidine, leucine, and lysine, along with cysteine, were damaged. However, loss of these amino acids became significant only when high concentrations of oxidants were present, e.g., 10 mM H_2_O_2_ or mixed 0.5 mM metmyoglobin/H_2_O_2_. Dorta, Ávila, Fuentes-Lemus, Fuentealba, and López-Alarcón [68] confirmed that methionine, cysteine, tyrosine, as well as tryptophan in MP were the most susceptible amino acid residues to peroxyl radicals.

As commonly observed, many of the oxidized amino acid side chain groups in MP are converted to carbonyl derivatives. Formation of protein-bound carbonyls takes place in virtually all muscle food systems, regardless of animal species or muscle types, i.e., fish [64,69], chicken [24,70], pork [9,71], or beef [72,73]. Two specific carbonyl compounds, α-aminoadipyl semialdehyde and γ-glutamyl semialdehyde, have been detected in oxidized meat [74]. Both are able to modify other amino acids producing Strecker degradation products [75]. Due to their electrophilic nature, protein carbonyls generated in meat products have the potential to bind with amine and sulfur groups to promote protein aggregation [39,76]. Likewise, the oxidative conversion of cysteine thiol groups to disulfide bonds plays an essential role in protein gelation and emulsification during meat processing [41]. Because myosin (the major protein in lean muscle tissue) has a particularly high sulfhydryl content (41 free cysteine residues per molecule), the amount of lost sulfhydryl groups is used to sensitively assess the extent of MP oxidation [77]. Other measurements have also been introduced to quantify ROS-induced muscle protein structural changes, e.g., differential scanning calorimetry, FTIR, circular dichroism, tryptophan fluorescence, and surface hydrophobicity [12,44,78]. In general, oxidatively stressed muscle proteins are of a less stable conformational structure and may exhibit molten globule characteristics. When structural modifications occur in the subfragment-2 (S2) “hinge” region of myosin, the formation of transglutaminase-catalyzed ε(γ-glutamyl)lysine bond at S2 is facilitated [79].

The oxidation-induced physicochemical changes have a profound impact on intermolecular interactions of MP as well as their reaction with other meat components, leading to both undesirable and desirable protein aggregation and functionality changes [80] and altered digestibility [81,82]. During postmortem aging, the inactivation of µ-calpain (a muscle endogenous protease) due to oxidation was thought to contribute to decreased beef tenderness [83]. Increased meat toughness and drip loss caused by freeze–thaw handling were also related to protein oxidation [84]. On the other hand, while oxidation is widely reported to impair functional properties of muscle proteins, many other investigations have shown beneficial effects of oxidation, e.g., improved gelling potential and emulsifying capacity [10,13,15]. The magnitude of such effects depends on the extent of modification of amino acid side chain groups and protein conformation and is influenced by the type of oxidizing agents present.

**Figure 2:**
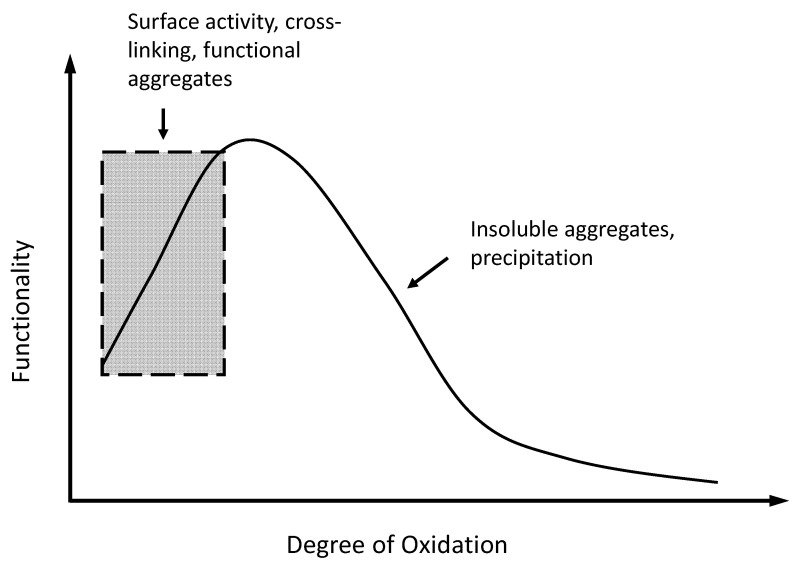
Functionality of oxidatively modified proteins. Functionality: gelation, emulsification, foaming, film formation, water-holding, etc.

A relationship between oxidation-induced structural changes and the aggregation and functionality (especially gel-forming properties) of MP has been reported in several studies [11,15,85]. The extent of protein structural modification has a profound influence on the gelling capacity of MP, hence, the final textural attributes (e.g., hardness, firmness, elasticity, and water-holding) of fabricated meat products [41]. For example, modified with an iron-catalyzed Fenton reaction that produces ^•^OH, a H_2_O_2_ dose-dependent structural modification and gelling behavior was established. The increased hardness of MP gels observed at low concentrations of H_2_O_2_ (<5 mM) was attributed to accentuated hydrophobic association and covalent interactions (disulfide and carbonyl-amine complex) at a level that favors an isotropic protein gel network rather than random aggregates [86]. Disulfide cross-linking of myosin on the side of rod (mostly light meromyosin) instead of the head (mostly heavy meromyosin) was conducive to protein matrix formation in MP gels [87,88]. As well, treatments with low concentrations of MDA (3 and 6 mM), a secondary product of lipid peroxidation, were shown to promote protein–protein association and significantly improve the emulsifying, gelling, and water-binding properties of MP gels [10]. Similarly, the incubation of MP with 2.5 mM MDA resulted in a MP-emulsion composite gel with improved elasticity, strength, and water-holding [85]. Based on these previous findings, a putative model can be put forth to summarize the oxidation-level effect (Figure 2). In essence, mild oxidative modification favors ordered protein–protein interactions, enabling a cohesive and elastic MP gel network, and strong oxidation predisposes MP to a weakened gelling ability due to the promotion of random aggregation and anisotropic network. Similar oxidation–functionality relationships have been observed in proteins from other food groups (discussed later), suggesting that the extent of surface and structural modification rather than the polypeptide type per se may be the principal causative factor.

**Figure 3:**
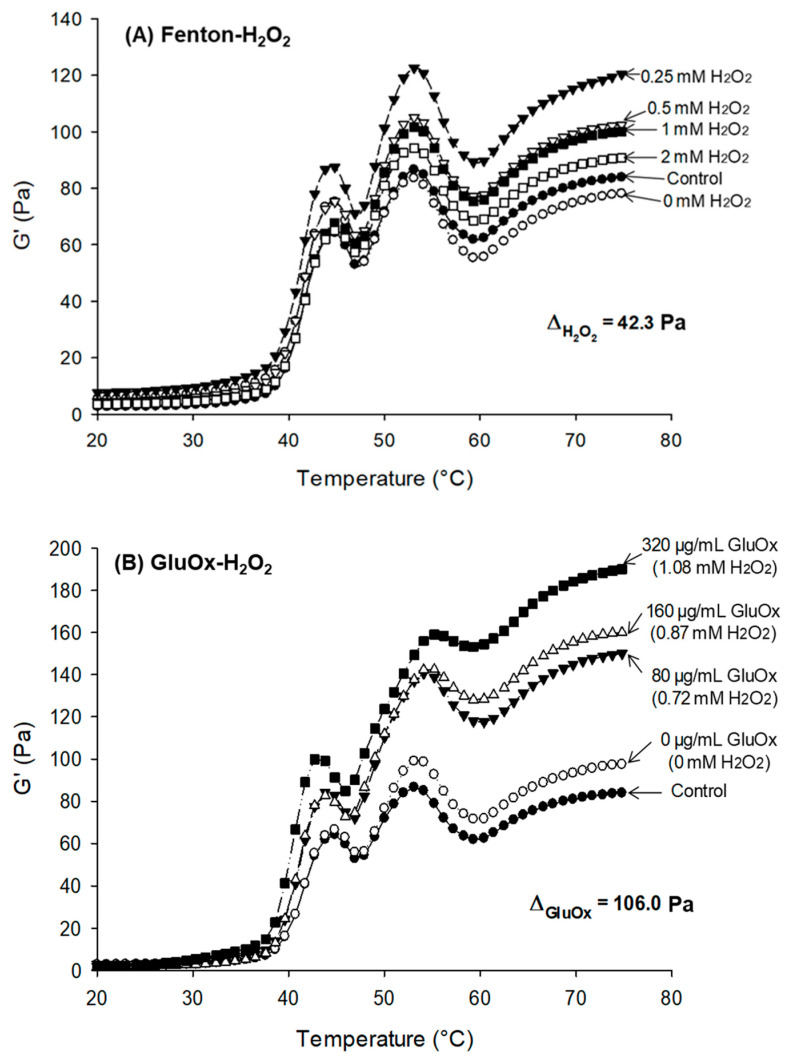
Storage modulus (G′) development of oxidatively modified myofibrillar protein during thermal gelation. (**A**) oxidation in a Fenton system (FeCl_2_/H_2_O_2_); (**B**) oxidation in a glucose oxidase (GluOx) system. ∆ denotes the differential between the highest and lowest final G′ values. Ref: Wang et al. [44].

For more progressive and controllable oxidation, Wang, Xiong, Sato, and Kumazawa [44] treated pork MP with mixed glucose and glucose oxidase (GluOx), which produces H_2_O_2_ for subsequent generation of ^•^OH. The catalytical H_2_O_2_ production proceeded linearly with the concentration of GluOx (80–320 µg/mL). Compared with the Fenton system with direct addition of 0.25, 0.5, 1, and 2 mM H_2_O_2_ to produce bursts of reactive ^•^OH, the enzymatic modification was mild and myosin aggregation therefore took place gradually. The progressive nature of H_2_O_2_ production, hence, ^•^OH, in the GluOx system explains the efficient and conducive protein cross-linking allowing a more elastic (G′) and stronger gel formed by the GluOx oxidation treatment (Figure 3). The enzymatic modification compared with direct chemical oxidation has the additional benefit of specificity, limiting the production of unintended by-products.

### 3.2. Legume Proteins

Most published legume protein oxidation studies have focused on soy protein and its major fractions. The studies conducted by Boatright and Hettiarachchy [22,89] resulted in some of the early publications on soy protein oxidation. The reports indicated a general trend of protein aggregation and solubility reduction when soy protein isolate (SPI) was exposed to residual lipids present in the protein preparation. A loss of sulfhydryls and formation of protein carbonyls were noted. Subsequently, Huang, Hua, and Qiu [90] induced soy protein oxidation in a model system comprised of lipoxygenase and linoleic acid, reporting that both disulfide and non-disulfide bonds were formed which played a significant role in protein aggregation. A further investigation showed that MDA was involved in the oxidative modification by the direct reaction with ε-amino (lysine) and sulfhydryl (cysteine) groups to generate carbonyl derivatives [91]. Covalent cross-linking as well as non-covalent interactions led to extensive aggregation and decreased solubility of soy proteins, of which β-conglycinin (7S) showed the greater sensitivity than glycinin (11S). The oxidative changes were generally detrimental to the functionality of soy proteins. For example, oxidation induced by peroxyl radical [derived from 2,2′-azobis (2-amidinopropane) dihydrochloride, or AAPH] resulted in dose-dependent reductions of water-holding capacity, gel hardness, and regularity of the interstices of the SPI gel [92]. The formation of random protein aggregates was thought to be a primary cause for the lost functionality. Although in these studies the researchers observed a general trend of decline in gelling properties of oxidatively stressed soy proteins, it is not clear whether an improved gelling performance would occur if the degree of oxidation was carefully controlled to certain low levels conducive to an isotropic protein network and gel microstructure, as often observed in MP gels.

On the other hand, when modified by hydroxyl radicals produced in the Fenton system with H_2_O_2_ concentrations up to 1.0 mM, Liu, Lu, Han, Chen, and Kong [93] noted significantly improved emulsifying activity of SPI. This was attributed to the exposures of hydrophobic amino acid residues and patches as well as the formation of soluble protein aggregates that appeared to contribute to the enhanced adsorption at the oil–water interface. The decline of emulsifying properties at higher H_2_O_2_ concentrations underscores the importance of controlling the extent of oxidative modification. These findings substantiated an early report [94] that structural modification of SPI by 0.05–1 mM AAPH improved the emulsifying activity and emulsion storage stability. More recently, Xu et al. [37] confirmed that moderate oxidation in an iron–ascorbate system with 1–15 mM H_2_O_2_ not only improved emulsifying properties of β-conglycinin but also reduced its allergenicity. It is worth noting that, because oxidation could both modify enzyme-accessible amino acid residues and alter the conformational structure to expose enzyme-susceptible peptide bonds, oxidation can result in either decreased or increased digestibility of soy proteins [23,94,95]. The degree of digestion is ultimately determined by the matrix structure and composition of affected proteins. The available contact area between the substrate (oxidized proteins) and proteolytic enzymes is essential to the overall effect of oxidation.

Compared with soy protein, other legume or pulse proteins have received minimal attention amongst the investigation of oxidative impact on functionality. Metal ion-catalyzed oxidation of pea proteins prior to O/W emulsion formation was found to increase the coalescence stability of emulsion droplets when compared with fresh pea protein [96]. This was explained by oxidation-induced protein fragmentation; however, protein conformational changes were likely involved as well. The interfacial film formed by oxidized protein was homogenous, in contrast to non-oxidized or mildly oxidized protein that formed a heterogeneous film structure.

### 3.3. Cereal Proteins

**Figure 4:**
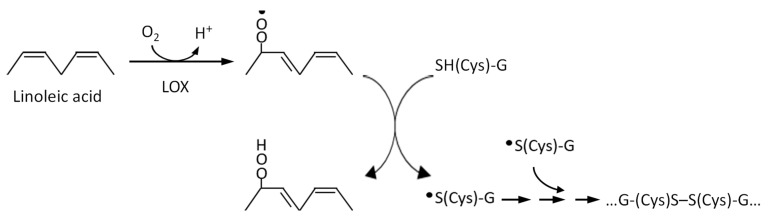
Inter-chain disulfide bond formation in glutenin (G) catalyzed by lipoxygenase (LOX).

Oxidation treatment of wheat dough with chemical oxidants and oxidative enzymes to improve the dough rheology and textural properties of baked foods has long been a commercial process [46]. The mechanism of textural improvement involves the oxidation of gluten thiol groups to form intermolecular disulfide bonds where SH/S–S exchange reactions take place (Figure 4). The ability of high-(HMW) and low-(LMW) molecular-weight subunits of glutenin to form a polymeric network through intermolecular disulfide bonds is essential to the unique rheological properties, mixing properties, and baking quality of wheat dough.

Hydrogen peroxide, ascorbic acid (dehydroascorbic acid), potassium bromate, lipoxyenase, and glucose oxidase are specific oxidizing agents for gluten modification and network formation [97]. When HMW glutenin subunits in wheat flour were treated with low concentrations of KBrO_3_, KIO_3_, and H_2_O_2_, polymers were formed via S–S bonds with H_2_O_2_ displaying the greatest effect [98]. Using 35S-labled radioactive glutathione, Koehler [99] was able to demonstrate that in ascorbate-treated wheat gluten, cysteine residues in the LMW subunits of glutenin were preferably converted to protein–protein disulfides contributing to the dough rheology and mixing performance.

Frazier, Brimblecombe, Daniels, and Russell Eggitt [100] applied soy lipoxygenase (LOX) to modify mechanical properties of doughs from wheat flours. The results indicated that the presence of PUFA as well air (oxygen) incorporation are fundamental requirements for the action of LOX. The deprivation of air in dough mixing or the addition of antioxidants inhibited the dough development. Lipid peroxyl radicals generated by the LOX catalysis reacted with sulfhydryls, leading to intermolecular S–S cross-linking to produce a viscoelastic protein network. Similar results were reported by Permyakova and Trufanov [101]. The addition of glucose oxidase, which produces H_2_O_2_ from glucose, increased the G′ and G″ of wheat dough [102]. The improved dough elasticity was noted by the reduction of tan δ (G″/G′). Bahrami, Bayliss, Chope, Penson, Perehinec, and Fisk [103] used atmospheric pressure cold plasma to modify the functionality of wheat flour. Treatments at 15 V (60 s) or 20 V (120 s) accelerated the production of hydroperoxides and head space n-hexanal from lipid oxidation resulting in protein oxidation as manifested by the formation of higher molecular weight polymers and an improved dough strength.

With a concentration of approximately 7–8%, rice proteins play an essential role in the pasting properties of rice and rice flour, whether refined or unrefined. Similar to wheat flour processing and wheat gluten utilization, controlling the extent of protein oxidation to promote pasting properties of rice flour has been a processing strategy in the production of rice-based products. For example, reducing agents, such as ascorbic acid and sodium sulfite, are capable of facilitating SH/S–S interchanges to promote the dough protein-starch network [104]. The texture of cooked rice grains was improved by both sodium sulfite and cysteine [105]. However, in rice grains, protein oxidation during storage is inevitable and could have a significant impact on the cooking and pasting properties of rice. Several recent studies have investigated the effect of oxidation on the structure and functionality of rice proteins. Using infrared and Raman spectroscopies, Guo et al. [106] demonstrated remarkable structural changes in the albumin, globulin, glutelin, and prolamin fractions in aged rice, namely, loss of α-helixes, burial of aliphatic amino acid side chains, and increase in the content of antiparallel β-sheets. Oxidation of sulfhydryl groups was implicated in the enhanced association between globulin and amylose (amylopectin) and weakened glutelin–starch interaction in the dough network system. These physicochemical changes were thought to be responsible for the gradual reduction of pasting properties in aged rice.

The occurrence of protein oxidation in rice during storage at 49 °C and under 85% relative humidity for up to 90 days was monitored by Shi, Wu, and Quan [28], and the impact on the rice gelatinization behavior was examined. The two major protein fractions, gliadin and glutelin, both displayed significant structural changes during storage. The formation of protein-bound carbonyls, the decrease in sulfur activity, and the increase in surface hydrophobicity in glutelin and, to a lesser extent, in gliadin, were shown to contribute to the loss of pasting properties, i.e., viscosity, elasticity, and strength of the dough. Packaging conditions, e.g., with or without vacuum, did not seem to prevent the loss of pasting properties associated with S–S cross-linking and aggregation of oryzenin [107]. On the other hand, the increase of the storage temperature, which promotes extensive oxidative cross-linking of oryzenin and impairs protein-starch interaction, always had a significant negative impact on the rice quality and contributed to the accelerated aging [108,109].

Zhou, Zhang, Zhao, Lin, Wang, and Wu [110] investigated the emulsifying properties of rice bran protein after the oxidation modification with AAPH, reporting a dose-dependent phenomenon. Mild oxidation with 0.2 mM (or less) AAPH was found to increase the protein surface hydrophobicity leading to an improved emulsifying capacity. With a higher degree of oxidation (>0.2 mM AAPH), soluble aggregates were formed from globulin, albumin, and glutelin. However, such hydrodynamic aggregation had a deleterious effect on the stability of prepared emulsions. Wu, Li, and Wu [111] treated mixed rice proteins with 0.1–1 mM 13-hydroperoxyoctadecadienoic acid (13-HPODE), a lipid peroxidation primary product formed during rice storage. Protein carbonyls were formed and sulfhydryl loss occurred along with the disruption of secondary structure (α-helix, β-turn, and random coil) and an increased β-sheet content. Water-holding capacity, foaming capacity, and foam stability were significantly reduced. However, at <0.1 mM 13-HPODE, emulsifying activity and emulsion stability of the mixed rice proteins were improved, which were attributed by the authors to the increased protein surface hydrophobicity and molecular flexibility. The same research group [112] also reported that treatments of rice proteins with 0.01–10 mM MDA led to a general decline in the secondary structure and surface hydrophobicity and an increase in the carbonyl content. The extensive aggregation involving both disulfide bonds and non-disulfide interactions between protein subunits resulted in the solubility reduction.

### 3.4. Milk Proteins

In milk, the bulk of total protein (~80%) is from casein, and whey proteins account for only ~20% of the total. However, due to their high solubility and remarkable functional properties (i.e., gelation and emulsification), whey proteins have been subjected to frequent investigations for oxidative changes. Under oxidative stress, whey protein isolate (WPI) has been shown to form heterogeneous aggregates dominated by disulfide bonds, and the impact of such oxidative modification on the protein functionality tends to be variable and inconsistent. For example, the dilatational rheology and viscoelasticity of the O/W emulsion interfacial membrane of WPI [113] and the mechanical properties (elasticity, rigidity, moisture permeability, etc.) and microstructural characteristics of whey protein films [20] were reportedly impaired by hydroxyl radicals. Similarly, despite the cross-linking to produce elongated protein aggregates, the rheology and network strength of WPI gels were shown to decrease with the progression of oxidation by ^•^OH [114]. However, both the emulsifying activity at neutral pH [114] and the gelling potential under alkaline conditions [18] were found to substantially increase following oxidative modification. Additionally, Tan, Wang, Chen, Niu, and Yu [115] observed improved gelling properties of O/W emulsions prepared with AAPH-oxidized WPI, attributing their findings to the tendency of fine emulsion droplets to aggregate into an immobile gel-like network that restricts the mobility of oil particles.

The results obtained from these studies may be difficult to compare since different oxidizing conditions were employed. Nevertheless, soluble protein particles produced by oxidizing stress, which have a propensity to develop an interactive protein matrix and cross-link at the oil-water interface, appear to have a major contribution in the reported functionality improvement. Hence, it may be stated that, as with the impact on muscle protein functionality, the oxidative modification can produce a nonmonotonic effect on whey protein functionality. That is, mild-to-moderate levels of oxidation, which causes partial unfolding and exposure of hydrophobic domains and other reactive groups, would enhance the gelling and emulsifying potential of whey proteins. Conversely, extensive oxidation induced by high dosages of oxidants would have a deleterious consequence. The substantial loss in protein solubility due to aggregation is often the dominant factor for the functionality losses, while the formation of surface-active and water-soluble aggregates generally leads to an improved functional performance of whey protein [116,117].

In addition to chemical oxidation, several physical methods have been tested for their efficacies to modify milk proteins. When subjecting WPI to UV radiation, Kristo, Hazizaj, and Corredig [118] showed that the UV treatment induced significant tertiary structural changes, tyrosine oxidation, an increased concentration of accessible sulfhydryl groups, protein aggregation, and improved digestibility. For β-casein and β-lactoglobulin, the presence of H_2_O_2_ and copper accelerated the UV-induced loss of tryptophan and increased the amount of oxidative products from both proteins [119], suggesting interactive effects. Segat et al. [120] used ozone processing to modify the chemical, structural, and functional properties of WPI. They found that ozonation increased protein structural flexibility without promoting disulfide bonds formation and improved the foaming capacity and foam stability of WPI.

Furthermore, due to the abundant presence of photosensitizers in milk, such as riboflavin and chlorophyll, photooxidation has received considerable attention in the study of milk protein oxidation and the effect on protein functionality. Mestdagh, Kerkaert, Cucu, and De Meulenaer [121] investigated light-induced oxidation of whey protein in the presence of the photosensitizer riboflavin. The formation of protein-lipid adducts noted in the process was linked to a decreased extractability of unsaturated lipids from the emulsion and large protein aggregate formation. Furthermore, such lipid and protein photosensitive co-oxidation decreased the digestibility of not only whey proteins but also casein present in O/W emulsions [122]. In the study of photooxidation of individual protein components in milk, Dalsgaard and Larsen [123] found that riboflavin-induced photooxidative modification of tryptophan and tyrosine residues changed the conformation of both α-casein and β-casein, leading to reduced digestibility by chymosin. Interestingly, β-lactoglobulin and lactoferrin, showing no significant conformational changes, became more proteolytically susceptible after photooxidation. In such photooxidative system, α-lactalbumin radicals were detected [124], and N-formylkynurenine and kynurenine (derived from tryptophan) as well as polymers of α-casein, β-casein, and lactoferrin were identified [21]. Dityrosine and ditryptophan were found to contribute to the cross-linking and aggregation of α-casein and β-casein induced by riboflavin-sensitized photooxidation especially under anaerobic conditions [125]. Milk protein oxidation induced by redox enzymes has also been investigated. For example, Østdal, Bjerrum, Pedersen, and Andersen [126] reported that radicals formed on lactoperoxidase by the reaction with H_2_O_2_ can be transferred onto β-lactoglobulin, bovine serum albumin, and caseins to produce long-lived protein radicals. Di-tyrosine, which has a potential role in milk protein aggregation, was formed in the presence of H_2_O_2_-activated endogenous or exogenous lactoperoxidase.

While deliberated aggregation to modify the functionality of milk proteins can be achieved by controlling the degree of oxidation, there are cases where oxidants are added to prevent protein aggregation. A special strategy is the use of hydrogen peroxide to react with the thiol groups in milk proteins to transform the sulfhydryl groups into sulfur derivatives (sulfenic, sulfinic, and sulfonic groups) so as to prevent S–S bond formation. Improved heat stability of β-lactoglobulin after the modification with H_2_O_2_ (molar ratio 1:2) was reported in the study of Krämer, Thulstrup, Lund, and Davies [127] where the lone free Cys121 residue was blocked. Sulfenic acid formation was confirmed by both immunoblotting and LC–MS/MS. For mixed whey proteins, treatments with 1.4–14% (v/v, protein basis) H_2_O_2_ produced a significant dose-dependent heat stability improvement, which was evidenced by the lack of appreciable aggregation of β-lactoglobulin and α-lactalbumin even at 120 °C when the control samples formed gels [128].

### 3.5. Egg Proteins

Egg proteins are divided into two major groups: the albumen (white) and yolk proteins. The albumen fraction is free of lipid, hence, oxidatively stable. The yolk fraction contains a large amount of lipids, but the vitellin proteins are generally stable due to the presence of natural antioxidants present in the yolk. Egg white (albumen) is valued for its excellent foaming and gelling (coagulating) properties, while egg yolk is commonly utilized as an O/W emulsifier. However, when used as formulation ingredients, egg proteins, similar to the proteins from other food commodity groups, can become susceptible to oxidation. Several researchers have investigated oxidants-induced structural and functional changes in egg white protein in different model systems. Wang et al. [17] explored the functionality of egg white upon exposure to ^•^OH generated by 0.1 mM FeCl_3_, 0.1 mM ascorbic acid, and different concentrations of H_2_O_2_. The treatment with H_2_O_2_ up to 5 mM significantly increased the emulsifying and foaming capacity of egg white, and the emulsions and foams also exhibited an improved stability. However, higher levels of H_2_O_2_ impaired both functional properties. Denaturation and aggregation, along with the oxidation of free thiol groups to disulfide bonds, were the common consequence of oxidative stress. The findings were corroborated by Duan et al. [129] who observed a similar oxidant concentration-dependent effect on the foaming properties of egg white. In the latter study, the authors treated egg white protein with AAPH, noticing increased foaming activity and foam stability at 0.2 mM AAPH. At much higher concentrations of AAPH (5 or 25 mM), although the foaming activity also showed an improvement, the resulting foam had a poor stability. The interfacial functionality of egg white protein has also been studied by Alavi et al. [130] using an ascorbate/H_2_O_2_ redox system. According to this study, the aggregates formed by oxidatively stressed protein played a major role in the formation and stabilization of the viscoelastic interfacial film. The improved physical stability against droplet coalescence (emulsion) and air bubble collapses (foam) was seen over a broad pH range.

The coagulation potential and gelation capacity of oxidized egg white have also been studied. Bao, Wu, Cheng, and Chi [131] modified egg albumin (the predominant protein in egg white) with AAPH. The oxidation treatment promoted protein carbonyl formation, sulfhydryl loss, structural destabilization, and aggregation. Low-to-moderate oxidizing conditions (0.04–1 mM AAPH) were found to enhance the gel strength of albumin, while oxidation at higher oxidant concentrations (3–5 mM AAPH) decreased gel strength as well as water-holding capacity. In a separate study reported by Geng, Huang, Huang, He, Li, and Ma [132], the incubation of egg ovalbumin with 0.1 mM FeCl_3_, 1 mM ascorbate, and 5 mM H_2_O_2_ resulted in the gradual loss of gelling capacity and was detrimental to water-holding capacity of the protein. Random aggregates were formed in the gel matrix when compared with the gel prepared with nonoxidized albumin. To avoid heat stress, Alavi, Momen, Emam-Djomeh, Salami, and Moosavi-Movahedi [133] proposed the application of ascorbate/H_2_O_2_ redox pair to modify egg white protein for cold-set gels. Protein aggregates formed at pH 10 and 11 were shown to spontaneously form gels without heat.

There is limited information on the potential influence of oxidation on the functionality of egg yolk protein. Bao, Kang, Xu, Sun, and Lin [134] applied AAPH to modify the structure of egg yolk high-density lipoprotein and examined the impact on emulsifying capacity. Based on the results, the authors concluded that moderate oxidizing conditions (up to 0.2 mM AAPH) enhanced emulsification while higher dosages of AAPH (up to 10 mM) interfered with protein deposition at the interface leading to decreased emulsifying activity. Methionine, cysteine, and tyrosine were found to be the main amino acids modified by AAPH. Furthermore, while oxidation under controlled conditions may have a positive influence on the functional properties of egg proteins, the potential health impact of egg protein oxidation remains poorly understood. A recent study showed that the allergenicity of egg albumin was actually enhanced following oxidative stress [135]. In this study, albumin samples were oxidized by 1, 5, 15, and 25 mM AAPH, and the exposure of allergenic epitopes was assessed. The structural unfolding and epitope exposures led to increased IgG/IgE binding ability suggesting the potential adverse effect on consumers who are hypersensitive to egg white.

## 4. Antioxidant Strategies for Modulating Protein Oxidation

### 4.1. Antioxidants Incorporated as Food Additives

The usefulness of oxidative modification to modify protein structure depends on the specific food applications, and the incorporation of antioxidants to manipulate protein oxidation is the simplest approach. As aforementioned, oxidation generally reduces protein solubility, hence, may be undesirable for protein-based beverages. However, limited oxidation could often promote protein aggregation and the generation of functional particles that play an important role in structure formation in solid and semi-solid foods through cross-linking, gelation, and interaction with lipids. To regulate protein oxidation, antioxidants are usually applied [4,136,137]. Boatright and Hettiarachchy [138] reported that the addition of 200 ppm (lipid basis) tert-butylhydroquinone (TBHQ) during SPI processing decreased the oxidative damage to free sulfhydryls by 32% and lowered the protein carbonyl content by 20%, leading to a 56% improvement of protein solubility. Although not directly targeting proteins, hydrophobic binding of resveratrol to SPI at the oil–water interface significantly improved the oxidative stability of O/W emulsions [139].

There have been considerable efforts to control the degree of oxidative modification using specific antioxidants to exploit the potential benefit of oxidation. For this purpose, most exploratory studies utilizing antioxidants for protein oxidation control have focused on muscle foods. In the preparation of surimi and surimi-like muscle protein concentrates, a combination of propyl gallate (radical scavenger) and sodium ascorbate (reducing agent) was applied in the washing solution to inhibit the oxidation of both lipids and proteins [14,64,140,141,142]. This antioxidant strategy inhibited lipid oxidation but increased the content of protein-bound carbonyls due to the binding with oxidized ascorbate (dehydroascorbate) resulting in significantly improved gelling potential and gel properties. This effect was somewhat similar to that of ascorbic acid (or dehydroascorbic acid) used in bakery processing to oxidatively facilitate disulfide bond formation between gluten polypeptides for improved dough performance and structural characteristics [98,143].

Due to their demonstrated efficacy for inhibiting lipid oxidation in food systems, plant phenolic compounds as a group of antioxidants have been increasingly studied for their potential to regulate protein oxidation and modify protein functionality [34,144,145]. Phenolic compounds can mediate protein interactions by acting as antioxidants or prooxidants depending on their chemical structure and concentration [146]. Unlike most other antioxidants, phytophenols and their oxidized quinone products have the ability to bind to proteins through both noncovalent (electrostatic attraction, hydrophobic stacking, van der Waals interactions, and hydrogen bond) and covalent (disulfide and carbonyl-amine adduction) forces [147,148]. Using triple TOF MS/MS, Tang et al. [149] identified rosmarinic acid–myosin heavy chain adducts in oxidatively stressed meat, which involved cysteine, lysine, arginine, and histidine side chain groups. Phenol–protein interactions can lead to substantial structural changes and alter the functional behavior of proteins, including gelation, emulsification, foaming, and film formation. Based on the intrinsic tryptophan fluorescence measurement, Cao and Xiong [150] showed that ^•^OH stress alone significantly destabilized MP conformation, and the presence of chlorogenic acid intensified oxidation-initiated loss of α-helix conformation and modified the tertiary structure of MP. Similarly, Jia, Wang, Shao, Liu, and Kong [151] claimed that catechin was effective in destabilizing the tertiary structure of MP and increased surface hydrophobicity.

In the study reported by Jongberg, Tørngren, Gunvig, Skibsted, and Lund [152], green tea and rosemary phenolic extracts (500 and 400 ppm, respectively) suppressed the production of TBARS and protein carbonyls in bologna-type sausage but promoted protein polymerization through quinone binding with thiol groups where protein-bound phenoxyl radicals were implicated. The same research groups showed that when packaged under a high-oxygen modified atmosphere, the accelerated loss of thiol groups in beef patties treated with white grape extract reduced myosin cross-linking presumably due to fewer free sulfhydryls for disulfide linkage formation [153]. Similarly, Estévez, Ventanas, and Cava [154] showed that the addition of rosemary essential oil (150, 300, and 600 ppm) inhibited lipid and protein oxidation and reduced the hardening of refrigerated frankfurters. Tang, Zhang, Dai, Li, Xu, and Zhou [155] used mass spectrometry to demonstrate that rosmarinic acid (RosA) conjugated with MP via adduction to cysteine residues (Cys949) in oxidatively stressed myosin.

Resulting from structural modification and conjugation with phenolic compounds, significant changes in the functionality of MP occur. Treatments of MP with 6 and 30 μmol/g chlorogenic acid significantly increased the viscoelasticity of MP gels while 150 μmol/g impaired the gelation due to excessive aggregation and insolubility [150]. Similarly, gallic acid and tea polyphenols have been shown to exert a dose-dependent effect on increasing the elasticity and hardness of MP gels [156,157]. Jia et al. [158] found that the elasticity of MP gels was increased by rutin in the concentrations range of 10–100 µmol/g. Furthermore, the type of amino acid residues modified is important. Tang et al. [159] indicated that low concentrations of RosA (0.05 and 0.25 mM) allowed RosA–cysteine and arginine/histidine–RosA–cysteine adductions, leading to a positive effect on the rheological attributes of the MP gels. The incorporation of 0.5–2 mM epigallocatechin gallate was effective in mitigating the loss of MP functionality caused by 12 mM MDA [10].

The effect of phenolic compounds on the functionality of MP has also been evaluated in enzyme-oxidizing systems. Under a moderate oxidizing condition induced by glucose oxidase, Guo and Xiong [34] observed substantially improved MP gelling capacity by 60 μmol gallic acid, i.e., up to 48% in storage modulus (G′) and 23% in gel strength. Similarly, squid ink tyrosinase, a polyphenol oxidase, was found to increase the gelling properties of fish surimi incubated with tannic acid [160]. Balange and Benjakul [161] examined the effect of tannins on the gelation of fish surimi protein with no added oxidants but at various pH (3, 7, 8, and 9), noting that alkaline pH significantly enhanced gel breaking force due to the interaction with quinone derivatives. When oxidized using ultraviolet irradiation (UVA), free radicals produced are capable of modifying the structure of MP, and the presence of phlorotannin extracts (PTE) further improved the MP gel strength by 75% [162].

### 4.2. Antioxidants Incorporated In Situ

*In situ* strategies to control protein oxidation in protein-based foods have been explored. Supplementation of animal feed with antioxidants, such as vitamin E and polyphenol-rich plant extracts, has been shown to increase the antioxidant potential and color stability of fresh beef [163,164] and also suppress the generation of protein carbonyls in postmortem meat for generally improved quality [72,165]. The protection of muscle protein against oxidation *in vivo* ensures water-holding capacity in cooked meat [166]. Feeding chickens antioxidant plant extracts (100 ppm rosemary, green tea, grape seed, and tomato extracts) was shown to improve the oxidative stability of muscle lipids but not proteins [167]. On the other hand, broiler chickens fed 200 ppm organic selenium (a cofactor of glutathione peroxidase) as a supplement to other antioxidative minerals displayed significantly less lipid oxidation (TBARS) in breast muscle during refrigerated storage [168]. Furthermore, protein oxidation (sulfhydryl and carbonyl) was partially inhibited, which corresponded to greater activity of glutathione peroxidase, catalase, and superoxide dismutase. The increased oxidative stability led to less purge loss and an improved cooking yield of the broiler meat [169]. Another *in situ* approach to increasing the selenium content in food is through legume production. The biofortification of selenium through the soybean production system was reported to increase the selenium content and improve the oxidative stability of soy β-conglycinin when exposed to peroxyl radical oxidative stress [170]. Both secondary and tertiary structures of β-conglycinin were protected by the enriched organic selenium. Although animal feeding and plant biofortification may be good strategies to enhance protein oxidative stability, the cost associated with such nutritional regimens can be disadvantageous and even prohibitive. For example, to be effective in retarding myoglobin and lipid oxidation in beef, thus, justifying nutrition supplementation in cattle feed, a minimum of 500 international units (IU) of vitamin E per day up to several months may be required [164]. Besides, muscle accumulates tocopherols and other antioxidants to different extents, and the threshold concentration varies between muscles, adding additional challenges to the *in situ* procedure.

## 5. Conclusions and Final Remarks

Protein oxidation, a lesser recognized phenomenon compared with lipid oxidation, occurs ubiquitously across commodity food groups during preparation, processing, and storage. Reactive oxygen species, including ^•^OH and RO^•^, many secondary products of lipid oxidation, as well as Strecker degradation carbonyls and ketones, are the major initiators of protein oxidation. Oxidative modification of amino acid side chain groups and structural changes (both secondary and tertiary) lead to aggregation and produce either functional or nonfunctional protein particles. The former are generally soluble and can be generated using mild oxidizing conditions, while the latter occur when proteins are extensively modified at high concentrations of or prolonged exposures to oxidants. Improved gelling, emulsifying, foaming, film-forming, water-holding, and texture-enhancing properties are possible with the application of appropriate natural chemical modifiers. Multifunctional food additives, e.g., ascorbic acid and phenolic acids, are particularly attractive due to their ability to simultaneously modify proteins and inhibit undesirable lipid oxidation. Oxidative enzymes, including glucose oxidase and lipoxyenase, are also promising candidates due to their mild action and progressive catalysis for ROS production.

There is a need to explore the optimum level(s) of oxidative modification using different ingredients and processing strategies and ensure that the results are batch-to-batch consistent and the product flavor is not compromised. To deliver nutritionally balanced and economically incentive dietary options, prepared foods are increasingly formulated with blends of different proteins. Although the surface and structural characteristics dictate the lability of proteins to oxidative modification [24], the preferred target polypeptide species in a concoction of proteins, e.g., plant protein-based meat analogues, could change, depending on the pH, salt concentration, temperature, and other structure-altering processing parameters. This is one of the areas where continuing research is warranted to fill in the knowledge gap. Finally, although mild oxidation may not have a significant impact on the nutritional quality of proteinaceous foods (since loss of essential amino acids and protein digestibility reduction tend to occur to a limited extent based on most published reports), the safety aspect associated with all levels of oxidation should be carefully investigated.

## Figures and Tables

**Table 1:** Examples of proteins from different sources that have been subjected to oxidation studies.

Food Source	Protein Studied	Functionality
Meat (pork, beef, rabbit, chicken, turkey)	Myofibrillar protein; myosin; gelatin	Gelation; emulsification; water-holding
Fish	Surimi (crude myofibrillar protein); gelatin	Gelation; film-forming
Milk	Whey protein; α-lactalbumin; β-lactoglobulin; lactoferrin; bovine serum albumin; caseins	Gelation; emulsification; film-forming
Egg	Egg white protein; ovalbumin; egg yolk protein	Foaming; gelation; water-holding; emulsification
Legume (soy, pea)	Protein isolate; β-conglycinin	Gelation; emulsification; water-holding
Cereal (wheat, rice)	Gluten; gliadin; glutenin; high-molecular-weight glutenin subunits; low-molecular-weight gluten subunits; oryzenin	Gluten network elasticity; water-holding; film-forming; emulsification

References are found within the body of the text.

**Table 2:** Oxidation of protein amino acid residue side chains and exemplary products.

Amino Acid	Products
Arginine	Glutamyl semialdehyde
Cysteine	Cystine; sulfenic acid; sulfinic acid; sulfonic acid
Glutamic acid	Oxalic acid
Histidine	2-oxohistidine; 4-hydroxyglutamate; aspartic acid
Leucine	3-, 4-, and 5-hydroxyleucine
Lysine	α-aminoadipyl semialdehyde
Methionine	Methionine sulfoxide; methione sulfone
Phenylalanine	2-, 3-, and 4-hydroxyphenylalanine; 2,3-dihydroxyphenylalanine
Proline	γ-glutamyl semialdehyde; 2-pyrrolidone; 4- and 5-hydroxyproline
Threonine	2-amino-3-ketobutyric acid
Tryptophan	2-, 4-, 5-, 6-, and 7-hydroxytryptophan; di-hydroxytryptophan, nitrotryptophan; 3-hydroxykynurenine, formalkynurenine
Tyrosine	Dityrosine; trityrosine; 3,4-dihydroxyphenylalanine

Ref: Butterfield and Stadtman [56]; Shacter [43]; Stadtman and Levine [51].

## References

[1] Frankel E.N. (1980). Lipid oxidation. Prog. Lipid Res..

[2] Stadtman E.R., Turner J.D., Beyreuther K., Theuring F. (1996). Protein aging and its relevance to the etiology of Alzheimer’s disease. Alzheimer’s Disease.

[3] Sohal R.S. (2002). Role of oxidative stress and protein oxidation in the aging process. Free Rad. Biol. Med..

[4] Estévez M., Xiong Y.L. (2019). Intake of oxidized proteins and amino acids and causative oxidative stress and disease: Recent scientific evidences and hypotheses. J. Food Sci..

[5] Roubal W.T., Tappel A.L. (1966). Polymerization of proteins induced by free-radical lipid peroxidation. Arch. Biochem. Biophys..

[6] Kanner J., Karel M. (1976). Changes in lysozyme due to reactions with peroxidizing methyl linoleate in a dehydrated model system. J. Agric. Food Chem..

[7] Schaich K.M., Pryor W.A. (1980). Free radical initiation in proteins and amino acids by ionizing and ultraviolet radiations and lipid oxidation—Part III: Free radical transfer from oxidizing lipids. Crit. Rev. Food Sci. Nutr..

[8] Funes J.A., Weiss U., Karel M. (1982). Effects of reaction conditions and reactant concentrations on polymerization of lysozyme reacted with peroxidizing lipids. J. Agric. Food Chem..

[9] Estévez M., Ventanas S., Cava R. (2007). Oxidation of lipids and proteins in frankfurters with different fatty acid compositions and tocopherol and phenolic contents. Food Chem..

[10] Lv Y., Chen L., Wu H., Xu X., Zhou G., Zhu B., Feng X. (2019). (-)-Epigallocatechin-3-gallate-mediated formation of myofibrillar protein emulsion gels under malondialdehyde-induced oxidative stress. Food Chem..

[11] Parkington J.K., Xiong Y.L., Blanchard S.P., Xiong S., Wang B., Srinivasan S., Froning G.W. (2000). Chemical and functional properties of oxidatively modified beef heart surimi stored at 2 °C. J. Food Sci..

[12] Sun W., Zhou F., Sun D.W., Zhao M. (2013). Effect of oxidation on the emulsifying properties of myofibrillar proteins. Food Bioproc. Technol..

[13] Srinivasan S., Hultin H.O. (1997). Chemical, physical, and functional properties of cod proteins modified by a nonenzymic free-radical-generating system. J. Agric. Food Chem..

[14] Wan L., Xiong Y.L., Decker E.A. (1993). Inhibition of oxidation during washing improves the functionality of bovine cardiac myofibrillar protein. J. Agric. Food Chem..

[15] Xiong Y.L., Blanchard S.P., Ooizumi T., Ma Y. (2010). Hydroxyl radical and ferryl-generating systems promote gel network formation of myofibrillar protein. J. Food Sci..

[16] Chang K.C., Marshall H.F., Satterlee L.D. (1982). Sulfur amino acid stability. Hydrogen peroxide treatment of casein, egg white, and soy isolate. J. Food Sci..

[17] Wang J., Zhao Y., Niu S., Wang X., Chen F. (2018). Effect of oxidation induced by hydroxyl radical-mediated model on molecular structural and physical character of egg white powder. Int. J. Food Sci. Technol..

[18] Alavi F., Momen S., Emam-Djomeh Z., Salami M., Moosavi-Movahedi A.A. (2018). Radical cross-linked whey protein aggregates as building blocks of non-heated cold-set gels. Food Hydrocoll..

[19] Marshall R.J. (1986). Effects of iodate, hydrogen peroxide and dichromate on the denaturation of whey proteins in heated milk. J. Dairy Res..

[20] Wang Y., Xiong Y.L., Rentfrow G.K., Newman M.C. (2013). Oxidation promotes cross-linking but impairs film-forming properties of whey proteins. J. Food Eng..

[21] Dalsgaard T.K., Otzen D., Nielsen J., Larsen L.B. (2007). Changes in structures of milk proteins upon photo-oxidation. J. Agric. Food Chem..

[22] Boatright W.L., Hettiarachchy N.S. (1995). Effect of lipids on soy protein isolate solubility. J. Am. Oil Chem. Soc..

[23] Duque-Estrada P., Berton-Carabin C.C., Nieuwkoop M., Dekkers B.L., Janssen A.E., van der Goot A.J. (2019). Protein oxidation and *in vitro* gastric digestion of processed soy-based matrices. J. Agric. Food Chem..

[24] Liu G., Xiong Y.L., Butterfield D.A. (2000). Chemical, physical, and gel-forming properties of oxidized myofibrils and whey- and soy-protein isolates. J. Food Sci..

[25] Wu W., Zhang C., Hua Y. (2009). Structural modification of soy protein by the lipid peroxidation product malondialdehyde. J. Sci. Food Agric..

[26] Cumbee B., Hildebrand D.F., Addo K. (1997). Soybean flour lipoxygenase isozymes effects on wheat flour dough rheological and breadmaking properties. J. Food Sci..

[27] Elkassabany M., Hoseney R.C. (1980). Ascorbic acid as an oxidant in wheat flour dough. II. Rheological effects. Cereal Chem..

[28] Shi J., Wu M., Quan M. (2017). Effects of protein oxidation on gelatinization characteristics during rice storage. J. Cereal Sci..

[29] Fu Q.Q., Liu R., Zhang W., Ben A., Wang R. (2020). *In vitro* susceptibility of oxidized myosin by μ-calpain or caspase-3 and the determination of the oxidation sites of myosin heavy chains. J. Agric. Food Chem..

[30] Lund M.N., Luxford C., Skibsted L.H., Davies M.J. (2008). Oxidation of myosin by haem proteins generates myosin radicals and protein cross-links. Biochem. J..

[31] Ostdal H., Andersen H.J., Davies M.J. (1999). Formation of long-lived radicals on proteins by radical transfer from heme enzymes—A common process?. Arch. Biochem. Biophys..

[32] Armenteros M., Morcuende D., Ventanas J., Estévez M. (2016). The application of natural antioxidants via brine injection protects Iberian cooked hams against lipid and protein oxidation. Meat Sci..

[33] Garcia-Lomillo J., González-SanJosé M.L., Skibsted L.H., Jongberg S. (2016). Effect of skin wine pomace and sulfite on protein oxidation in beef patties during high oxygen atmosphere storage. Food Bioproc. Technol..

[34] Guo A., Xiong Y.L. (2019). Glucose oxidase promotes gallic acid-myofibrillar protein interaction and thermal gelation. Food Chem..

[35] Wang X., Xiong Y.L., Sato H. (2017). Rheological enhancement of pork myofibrillar protein–lipid emulsion composite gels via glucose oxidase oxidation/transglutaminase cross-linking pathway. J. Agric. Food Chem..

[36] Ooms N., Delcour J.A. (2019). How to impact gluten protein network formation during wheat flour dough making. Curr. Opin. Food Sci..

[37] Xu J., Chen Z., Han D., Li Y., Sun X., Wang Z., Jin H. (2017). Structural and functional properties changes of β-conglycinin exposed to hydroxyl radical-generating systems. Molecules.

[38] Zhou F., Sun W., Zhao M. (2015). Controlled formation of emulsion gels stabilized by salted myofibrillar protein under malondialdehyde (MDA)-induced oxidative stress. J. Agric. Food Chem..

[39] Xiong Y.L., Decker E.A., Faustman C., Lopez-Bote C.J. (2000). Protein oxidation and implications for muscle food quality. Antioxidants in Muscle Foods: Nutritional Strategies to Improve Quality.

[40] Zhang W., Xiao S., Ahn D.U. (2013). Protein oxidation: Basic principles and implications for meat quality. Crit. Rev. Food Sci. Nutr..

[41] Xiong Y.L. (1996). Impacts of oxidation on muscle protein functionality. Proc. Recipr. Meat Conf..

[42] Lund M.N., Heinonen M., Baron C.P., Estévez M. (2011). Protein oxidation in muscle foods: A review. Mol. Nutr. Food Res..

[43] Shacter E. (2000). Quantification and significance of protein oxidation in biological samples. Drug Metab. Rev..

[44] Wang X., Xiong Y.L., Sato H., Kumazawa Y. (2016). Controlled cross-linking with glucose oxidase for the enhancement of gelling potential of pork myofibrillar protein. J. Agric. Food Chem..

[45] Xu F. (1996). Oxidation of phenols, anilines, and benzenethiols by fungal laccases: Correlation between activity and redox potentials as well as halide inhibition. Biochemistry.

[46] Hayward S., Cilliers T., Swart P. (2017). Lipoxygenases: From isolation to application. Comp. Rev. Food Sci. Food Saf..

[47] Kussendrager K.D., van Hooijdonk A.C.M. (2000). Lactoperoxidase: Physico-chemical properties, occurrence, mechanism of action and applications. Br. J. Nutr..

[48] DeRosa M.C., Crutchley R.J. (2002). Photosensitized singlet oxygen and its applications. Coord. Chem. Rev..

[49] Pattison D.I., Rahmanto A.S., Davies M.J. (2012). Photo-oxidation of proteins. Photochem. Photobiol. Sci..

[50] Halliwell B., Gutteridge J.M. (2015). Free Radicals in Biology and Medicine.

[51] Stadtman E.R., Levine R.L. (2003). Free radical-mediated oxidation of free amino acids and amino acid residues in proteins. Amino Acids.

[52] Welch K.D., Davis T.Z., van Eden M.E., Aust S.D. (2002). Deleterious iron-mediated oxidation of biomolecules. Free Rad. Biol. Med..

[53] Stadtman E.R., Berlett B.S. (1997). Reactive oxygen-mediated protein oxidation in aging and disease. Chem. Res. Toxicol..

[54] Van Bergen L.A., Roos G., de Proft F. (2014). From thiol to sulfonic acid: Modeling the oxidation pathway of protein thiols by hydrogen peroxide. J. Phys. Chem..

[55] Grimsrud P.A., Xie H., Griffin T.J., Bernlohr D.A. (2008). Oxidative stress and covalent modification of protein with bioactive aldehydes. J. Biol. Chem..

[56] Butterfield D.A., Stadtman E.R., Timiras P.S., Bittar E.E. (1997). Protein oxidation processes in aging brain. Advances in Cell Aging and Gerontology.

[57] Zhao J., Chen J., Zhu H., Xiong Y.L. (2012). Mass spectrometric evidence of malonaldehyde and 4-hydroxynonenal adductions to radical scavenging soy peptides. J. Agric. Food Chem..

[58] Levine R.L., Williams J.A., Stadtman E.R., Shacter E. (1994). Carbonyl assays for determination of oxidatively modified proteins. Methods Enzymol..

[59] Brady O.L., Elsmie G.V. (1926). The use of 2: 4-dinitrophenylhydrazine as a reagent for aldehydes and ketones. Analyst.

[60] Baraibar M.A., Ladouce R., Friguet B. (2013). Proteomic quantification and identification of carbonylated proteins upon oxidative stress and during cellular aging. J. Proteom..

[61] Uchiyama S., Inaba Y., Kunugita N. (2011). Derivatization of carbonyl compounds with 2,4-dinitrophenylhydrazine and their subsequent determination by high-performance liquid chromatography. J. Chromatogr. B Biomed. Appl..

[62] Buss H., Chan T.P., Sluis K.B., Domigan N.M., Winterbourn C.C. (1997). Protein carbonyl measurement by a sensitive ELISA method. Free Radic. Biol. Med..

[63] Kelleher S.D., Hultin H.O., Wilhelm K.A. (1994). Stability of mackerel surimi prepared under lipid-stabilizing processing conditions. J. Food Sci..

[64] Srinivasan S., Hultin H.O. (1995). Hydroxyl radical modification of fish muscle proteins. J. Food Biochem..

[65] Xiong Y.L., Decker E.A., Robe G.H., Moody W.G. (1993). Gelation of crude myofibrillar protein isolated from beef heart under antioxidative conditions. J. Food Sci..

[66] Granger D.N., Kvietys P.R. (2015). Reperfusion injury and reactive oxygen species: The evolution of a concept. Redox Biol..

[67] Park D., Xiong Y.L. (2007). Oxidative modification of amino acids in porcine myofibrillar protein isolates exposed to three oxidizing systems. Food Chem..

[68] Dorta E., Ávila F., Fuentes-Lemus E., Fuentealba D., López-Alarcón C. (2019). Oxidation of myofibrillar proteins induced by peroxyl radicals: Role of oxidizable amino acids. Food Res. Int..

[69] Nikoo M., Benjakul S., Yasemi M., Gavlighi H.A., Xu X. (2019). Hydrolysates from rainbow trout (*Oncorhynchus mykiss*) processing by-product with different pretreatments: Antioxidant activity and their effect on lipid and protein oxidation of raw fish emulsion. LWT.

[70] Soyer A., Ozalp B., Dalmış U., Bilgin V. (2010). Effects of freezing temperature and duration of frozen storage on lipid and protein oxidation in chicken meat. Food Chem..

[71] Liu Z., Xiong Y.L., Chen J. (2011). Morphological examinations of oxidatively stressed pork muscle and myofibrils upon salt marination and cooking to elucidate the water-binding potential. J. Agric. Food Chem..

[72] Mercier Y., Gatellier P., Renerre M. (2004). Lipid and protein oxidation *in vitro*, and antioxidant potential in meat from Charolais cows finished on pasture or mixed diet. Meat Sci..

[73] Srinivasan S., Xiong Y.L. (1996). Gelation of beef heart surimi as affected by antioxidants. J. Food Sci..

[74] Utrera M., Morcuende D., Rodríguez-Carpena J.G., Estévez M. (2011). Fluorescent HPLC for the detection of specific protein oxidation carbonyls–α-aminoadipic and γ-glutamic semialdehydes–in meat systems. Meat Sci..

[75] Estévez M., Ventanas S., Heinonen M. (2011). Formation of Strecker aldehydes between protein carbonyls–α-aminoadipic and γ-glutamic semialdehydes–and leucine and isoleucine. Food Chem..

[76] Estévez M. (2011). Protein carbonyls in meat systems: A review. Meat Sci..

[77] Srinivasan S., Xiong Y.L. (1997). Sulfhydryls in antioxidant-washed beef heart surimi. J. Muscle Foods.

[78] Liu C., Xiong Y.L. (2015). Oxidation-initiated myosin subfragment cross-linking and structural instability differences between white and red muscle fiber types. J. Food Sci..

[79] Li C., Xiong Y.L. (2019). Mild oxidation promotes myosin S2 cross-linking by microbial transglutaminase. Food Chem..

[80] Xiong Y.L., Decker E.A. (1995). Alterations of muscle protein functionality by oxidative and antioxidative processes. J. Muscle Foods.

[81] Liu G., Xiong Y.L. (2000). Electrophoretic pattern, thermal denaturation, and *in vitro* digestibility of oxidized myosin. J. Agric. Food Chem..

[82] Morzel M., Gatellier P., Sayd T., Renerre M., Laville E. (2006). Chemical oxidation decreases proteolytic susceptibility of skeletal muscle myofibrillar proteins. Meat Sci..

[83] Rowe L.J., Maddock K.R., Lonergan S.M., Huff-Lonergan E. (2004). Oxidative environments decrease tenderization of beef steaks through inactivation of μ-calpain. J. Anim. Sci..

[84] Xia X., Kong B., Liu Q., Liu J. (2009). Physicochemical change and protein oxidation in porcine longissimus dorsi as influenced by different freeze–thaw cycles. Meat Sci..

[85] Zhou F., Zhao M., Su G., Cui C., Sun W. (2014). Gelation of salted myofibrillar protein under malondialdehyde-induced oxidative stress. Food Hydrocoll..

[86] Xiong Y.L., Park D., Ooizumi T. (2009). Variation in the cross-linking pattern of porcine myofibrillar protein exposed to three oxidative environments. J. Agric. Food Chem..

[87] Ooizumi T., Xiong Y.L. (2006). Identification of cross-linking site(s) of myosin heavy chains in oxidatively stressed chicken myofibrils. J. Food Sci..

[88] Ooizumi T., Xiong Y.L. (2008). Hydroxyl radical oxidation destabilizes subfragment-1 but not the rod of myosin in chicken myofibrils. Food Chem..

[89] Boatright W.L., Hettiarachchy N.S. (1995). Lipid components that reduce protein solubility of soy protein isolates. J. Am. Oil Chem. Soc..

[90] Huang Y., Hua Y., Qiu A. (2006). Soybean protein aggregation induced by lipoxygenase catalyzed linoleic acid oxidation. Food Res. Int..

[91] Wu W., Zhang C., Kong X., Hua Y. (2009). Oxidative modification of soy protein by peroxyl radicals. Food Chem..

[92] Wu W., Hua Y., Lin Q., Xiao H. (2011). Effects of oxidative modification on thermal aggregation and gel properties of soy protein by peroxyl radicals. Int. J. Food Sci..

[93] Liu Q., Lu Y., Han J., Chen Q., Kong B. (2015). Structure-modification by moderate oxidation in hydroxyl radical-generating systems promote the emulsifying properties of soy protein isolate. Food Struct..

[94] Chen N., Zhao M., Sun W., Ren J., Cui C. (2013). Effect of oxidation on the emulsifying properties of soy protein isolate. Food Res. Int..

[95] Zhao J., Su G., Zhao M., Sun W. (2019). Physicochemical changes and *in vitro* gastric digestion of modified soybean protein induced by lipoxygenase catalyzed linoleic acid oxidation. J. Agric. Food Chem..

[96] Hinderink E.B., Kaade W., Sagis L., Schroën K., Berton-Carabin C.C. (2020). Microfluidic investigation of the coalescence susceptibility of pea protein-stabilised emulsions: Effect of protein oxidation level. Food Hydrocoll..

[97] Lucas I., Becker T., Jekle M. (2018). Gluten polymer networks—A microstructural classification in complex systems. Polymers.

[98] Veraverbeke W.S., Larroque O.R., Békés F., Delcour J.A. (2000). *In vitro* polymerization of wheat glutenin subunits with inorganic oxidizing agents. I. Comparison of single-step and stepwise oxidations of high molecular weight glutenin subunits. Cereal Chem..

[99] Koehler P. (2003). Effect of ascorbic acid in dough: Reaction of oxidized glutathione with reactive thiol groups of wheat glutelin. J. Agric. Food Chem..

[100] Frazier P.J., Brimblecombe F.A., Daniels N.W., Russell Eggitt P.W. (1977). The effect of lipoxygenase action on the mechanical development of doughs from fat-extracted and reconstituted wheat flours. J. Sci. Food Agric..

[101] Permyakova M.D., Trufanov V.A. (2011). Effect of soybean lipoxygenase on baking properties of wheat flour. Appl. Biochem. Microbiol..

[102] Miller K.A., Hoseney R.C. (1999). Dynamic rheological properties of wheat starch-gluten doughs. Cereal Chem..

[103] Bahrami N., Bayliss D., Chope G., Penson S., Perehinec T., Fisk I.D. (2016). Cold plasma: A new technology to modify wheat flour functionality. Food Chem..

[104] Guo Y., Tu K., Pan L., Zhang W., Zhang Y. (2012). Effects of three reducing agents on pasting properties of stored rice. Starch/Stärke.

[105] Ohno T., Tomatsu M., Toeda K., Ohisa N. (2007). Texture of cooked rice prepared from aged rice and its improvement by reducing agents. Biosci. Biotechnol. Biochem..

[106] Guo Y., Cai W., Tu K., Tu S., Wang S., Zhu X., Zhang W. (2013). Infrared and Raman spectroscopic characterization of structural changes in albumin, globulin, glutelin, and prolamin during rice aging. J. Agric. Food Chem..

[107] Tananuwong K., Malila Y. (2011). Changes in physicochemical properties of organic hulled rice during storage under different conditions. Food Chem..

[108] Chrastil J. (1990). Protein-starch interactions in rice grains. Influence of storage on oryzenin and starch. J. Agric. Food Chem..

[109] Thanathornvarakul N., Anuntagool J., Tananuwong K. (2016). Aging of low and high amylose rice at elevated temperature: Mechanism and predictive modeling. J. Cereal Sci..

[110] Zhou L., Zhang Y., Zhao C., Lin H., Wang Z., Wu F. (2017). Structural and functional properties of rice bran protein oxidized by peroxyl radicals. Int. J. Food Prop..

[111] Wu X., Li F., Wu W. (2020). Effects of oxidative modification by 13-hydroperoxyoctadecadienoic acid on the structure and functional properties of rice protein. Food Res. Int..

[112] Li F., Wu X.J., Wu W. (2020). Effects of malondialdehyde-induced protein oxidation on the structural characteristics of rice protein. Int. J. Food Sci. Technol..

[113] Berton-Carabin C.C., Schröder A., Rovalino-Cordova A., Schroën K., Sagis L. (2016). Protein and lipid oxidation affect the viscoelasticity of whey protein layers at the oil–water interface. Eur. J. Lipid Sci. Technol..

[114] Kong B., Xiong Y.L., Cui X., Zhao X. (2013). Hydroxyl radical-stressed whey protein isolate: Functional and rheological properties. Food Bioproc. Technol..

[115] Tan Y., Wang J., Chen F., Niu S., Yu J. (2016). Effect of protein oxidation on kinetics of droplets stability probed by microrheology in O/W and W/O emulsions of whey protein concentrate. Food Res. Int..

[116] Cui X., Xiong Y.L., Kong B., Zhao X., Liu N. (2012). Hydroxyl radical-stressed whey protein isolate: Chemical and structural properties. Food Bioproc. Technol..

[117] Feng X., Li C., Ullah N., Cao J., Lan Y., Ge W., Hackman R.M., Li Z., Chen L. (2015). Susceptibility of whey protein isolate to oxidation and changes in physicochemical, structural, and digestibility characteristics. J. Dairy Sci..

[118] Kristo E., Hazizaj A., Corredig M. (2012). Structural changes imposed on whey proteins by UV irradiation in a continuous UV light reactor. J. Agric. Food Chem..

[119] Scheidegger D., Larsen G., Kivatinitz S.C. (2016). Oxidative consequences of UV irradiation on isolated milk proteins: Effects of hydrogen peroxide and bivalent metal ions. Int. Dairy J..

[120] Segat A., Misra N.N., Fabbro A., Buchini F., Lippe G., Cullen P.J., Innocente N. (2014). Effects of ozone processing on chemical, structural and functional properties of whey protein isolate. Food Res. Int..

[121] Mestdagh F., Kerkaert B., Cucu T., de Meulenaer B. (2011). Interaction between whey proteins and lipids during light-induced oxidation. Food Chem..

[122] Obando M., Papastergiadis A., Li S., de Meulenaer B. (2015). Impact of lipid and protein co-oxidation on digestibility of dairy proteins in oil-in-water (O/W) emulsions. J. Agric. Food Chem..

[123] Dalsgaard T.K., Larsen L.B. (2009). Effect of photo-oxidation of major milk proteins on protein structure and hydrolysis by chymosin. Int. Dairy J..

[124] Dalsgaard T.K., Triquigneaux M., Deterding L., Summers F., Ranguelova K., Mortensen G., Mason R.P. (2013). Site-specific detection of radicals on α-lactalbumin after a riboflavin-sensitized reaction, detected by immuno-spin trapping, ESR, and MS. J. Agric. Food Chem..

[125] Fuentes-Lemus E., Silva E., Leinisch F., Dorta E., Lorentzen L.G., Davies M.J., Lopez-Alarcon C. (2018). α- and β-Casein aggregation induced by riboflavin-sensitized photo-oxidation occurs via di-tyrosine cross-links and is oxygen concentration dependent. Food Chem..

[126] Østdal H., Bjerrum M.J., Pedersen J.A., Andersen H.J. (2000). Lactoperoxidase-induced protein oxidation in milk. J. Agric. Food Chem..

[127] Krämer A.C., Thulstrup P.W., Lund M.N., Davies M.J. (2016). Key role of cysteine residues and sulfenic acids in thermal-and H_2_O_2_-mediated modification of β-lactoglobulin. Free Radcial Biol. Med..

[128] Sutariya S., Patel H. (2017). Effect of hydrogen peroxide on improving the heat stability of whey protein isolate solutions. Food Chem..

[129] Duan X., Li M., Shao J., Chen H., Xu X., Jin Z., Liu X. (2018). Effect of oxidative modification on structural and foaming properties of egg white protein. Food Hydrocoll..

[130] Alavi F., Emam-Djomeh Z., Momen S., Mohammadian M., Salami M., Moosavi-Movahedi A.A. (2019). Effect of free radical-induced aggregation on physicochemical and interface-related functionality of egg white protein. Food Hydrocoll..

[131] Bao Z.J., Wu J.P., Cheng Y., Chi Y.J. (2017). Effects of lipid peroxide on the structure and gel properties of ovalbumin. Process. Biochem..

[132] Geng F., Huang Y., Huang Q., He D., Li S., Ma M. (2018). Effect of hydroxyl radical-induced oxidation on the structure and heat-induced gel properties of ovalbumin. J. Food Process. Preserv..

[133] Alavi F., Momen S., Emam-Djomeh Z., Salami M., Moosavi-Movahedi A.A. (2018). Tailoring egg white proteins by a GRAS redox pair for production of cold-set gel. LWT.

[134] Bao Z., Kang D., Xu X., Sun N., Lin S. (2019). Variation in the structure and emulsification of egg yolk high-density lipoprotein by lipid peroxide. J. Food Biochem..

[135] Zhang J.J., Tu Z.C., Wang H., Hu Y.M., Du P.C., Yang Y.P. (2020). Mechanism of the effect of 2, 2′-azobis (2-amidinopropane) dihydrochloride simulated lipid oxidation on the IgG/IgE binding ability of ovalbumin. Food Chem..

[136] Jongberg S., Lund M.N., Skibsted L.H., Barbosa-Cánovas G., Pastore G., Candoğan K., Medina Meza I.G., Caetano Da Silva Lannes S., Buckle K., Yada R., Rosenthal A. (2017). Protein oxidation in meat and meat products. Challenges for antioxidative protection. Global Food Security and Wellness.

[137] Xiong Y.L., Mine Y., Li-Chan E., Jiang B. (2010). Antioxidant peptides. Bioactive Proteins and Peptides as Functional Foods and Nutraceuticals.

[138] Boatright W.L., Hettiarachchy N.S. (1995). Soy protein isolate solubility and surface hydrophobicity as affected by antioxidants. J. Food Sci..

[139] Wan Z.L., Wang J.M., Wang L.Y., Yuan Y., Yang X.Q. (2014). Complexation of resveratrol with soy protein and its improvement on oxidative stability of corn oil/water emulsions. Food Chem..

[140] Srinivasan S., Xiong Y.L., Decker E.A. (1996). Inhibition of protein and lipid oxidation in beef heart surimi-like material by antioxidants and combinations of pH, NaCl, and buffer type in the washing media. J. Agric. Food Chem..

[141] Wang B.W., Xiong Y.L., Srinivasan S. (1997). Chemical stability of antioxidant-washed beef heart surimi during frozen storage. J. Food Sci..

[142] Wang B., Xiong Y.L. (1998). Functional stability of antioxidant-washed, cryoprotectant-treated beef heart surimi during frozen storage. J. Food Sci..

[143] Grosch W., Wieser H. (1999). Redox reactions in wheat dough as affected by ascorbic acid. J. Cereal Sci..

[144] Utrera M., Morcuende D., Ganhão R., Estévez M. (2015). Role of phenolics extracting from Rosa canina L. on meat protein oxidation during frozen storage and beef patties processing. Food Bioproc. Technol..

[145] Quan T.H., Benjakul S., Sae-leaw T., Balange A.K., Maqsood S. (2019). Protein–polyphenol conjugates: Antioxidant property, functionalities and their applications. Trends Food Sci. Technol..

[146] Estévez M., Kylli P., Puolanne E., Kivikari R., Heinonen M. (2008). Oxidation of skeletal muscle myofibrillar proteins in oil-in-water emulsions: Interaction with lipids and effect of selected phenolic compounds. J. Agric. Food Chem..

[147] Ozdal T., Capanoglu E., Altay F. (2013). A review on protein–phenolic interactions and associated changes. Food Res. Int..

[148] Rohn S., Melton L., Shahidi F., Varelis P. (2019). Covalent interactions between proteins and phenolic compounds. Encyclopedia of Food Chemistry.

[149] Tang C.B., Zhang W.G., Wang Y.S., Xing L.J., Xu X.L., Zhou G.H. (2016). Identification of rosmarinic acid-adducted sites in meat proteins in a gel model under oxidative stress by triple TOF MS/MS. J. Agric. Food Chem..

[150] Cao Y., Xiong Y.L. (2015). Chlorogenic acid-mediated gel formation of oxidatively stressed myofibrillar protein. Food Chem..

[151] Jia N., Wang L., Shao J., Liu D., Kong B. (2017). Changes in the structural and gel properties of pork myofibrillar protein induced by catechin modification. Meat Sci..

[152] Jongberg S., Tørngren M.A., Gunvig A., Skibsted L.H., Lund M.N. (2013). Effect of green tea or rosemary extract on protein oxidation in Bologna type sausages prepared from oxidatively stressed pork. Meat Sci..

[153] Jongberg S., Skov S.H., Tørngren M.A., Skibsted L.H., Lund M.N. (2011). Effect of white grape extract and modified atmosphere packaging on lipid and protein oxidation in chill stored beef patties. Food Chem..

[154] Estévez M., Ventanas S., Cava R. (2005). Protein oxidation in frankfurters with increasing levels of added rosemary essential oil: Effect on color and texture deterioration. J. Food Sci..

[155] Tang C., Zhang W., Dai C., Li H., Xu X., Zhou G. (2015). Identification and quantification of adducts between oxidized rosmarinic acid and thiol compounds by UHPLC-LTQ-Orbitrap and MALDI-TOF/TOF tandem mass spectrometry. J. Agric. Food Chem..

[156] Cao Y., True A.D., Chen J., Xiong Y.L. (2016). Dual role (anti- and pro-oxidant) of gallic acid in mediating myofibrillar protein gelation and gel *in vitro* digestion. J. Agric. Food Chem..

[157] Li X., Liu C., Wang J., Li W., Lin B., Zhu W., Xu Y., Yi S., Mi H., Li J. (2020). Tea polyphenols affect oxidative modification and solution stability of myofibrillar protein from grass carp (*Ctenopharyngodon idellus*). Food Biophys..

[158] Jia N., Zhang F., Liu Q., Wang L., Lin S., Liu D. (2019). The beneficial effects of rutin on myofibrillar protein gel properties and related changes in protein conformation. Food Chem..

[159] Tang C.B., Zhang W.G., Zou Y.F., Xing L.J., Zheng H.B., Xu X.L., Zhou G.H. (2017). Influence of RosA-protein adducts formation on myofibrillar protein gelation properties under oxidative stress. Food Hydrocoll..

[160] Vate N.K., Benjakul S. (2016). Effect of the mixtures of squid ink tyrosinase and tannic acid on properties of sardine surimi gel. J. Food Sci. Technol..

[161] Balange A.K., Benjakul S. (2011). Effect of kiam wood extract as influenced by pH during oxygenation on mackerel surimi gel. J. Food Biochem..

[162] Jiang D., Shen P., Pu Y., Jin M., Yu C., Qi H. (2020). Enhancement of gel properties of Scomberomorus niphonius myofibrillar protein using phlorotannin extracts under UVA irradiation. J. Food Sci..

[163] Delosière M., Durand D., Bourguet C., Terlouw E.C. (2020). Lipid oxidation, pre-slaughter animal stress and meat packaging: Can dietary supplementation of vitamin E and plant extracts come to the rescue?. Food Chem..

[164] Faustman C., Chan W.K.M., Schaefer D.M., Havens A. (1998). Beef color update: The role for vitamin E. J. Anim. Sci..

[165] Mercier Y., Gatellier P., Viau M., Remignon H., Renerre M. (1998). Effect of dietary fat and vitamin E on color stability and on lipid and protein oxidation in turkey meat during storage. Meat Sci..

[166] Rowe L.J., Maddock K.R., Lonergan S.M., Huff-Lonergan E. (2004). Influence of early postmortem protein oxidation on beef quality. J. Anim. Sci..

[167] Smet K., Raes K., Huyghebaert G., Haak L., Arnouts S., de Smet S. (2008). Lipid and protein oxidation of broiler meat as influenced by dietary natural antioxidant supplementation. Poult. Sci..

[168] Delles R.M., Xiong Y.L., True A.D., Ao T., Dawson K.A. (2014). Dietary antioxidant supplementation enhances lipid and protein oxidative stability of chicken broiler meat through promotion of antioxidant enzyme activity. Poult. Sci..

[169] Delles R.M., Xiong Y.L., True A.D., Ao T., Dawson K.A. (2015). Augmentation of water-holding and textural properties of breast meat from oxidatively stressed broilers through dietary antioxidant regimens. Br. J. Poult. Sci..

[170] Zhao X., Zhao Q., Chen H., Xiong H. (2019). Distribution and effects of natural selenium in soybean proteins and its protective role in soybean β-conglycinin (7S globulins) under AAPH-induced oxidative stress. Food Chem..

